# Supplementary material for: Dietary Black Soldier Fly (Hermetia illucens)—Dipterose-BSF—Enhanced Zebrafish Innate Immunity Gene Expression and Resistance to Edwardsiella tarda Infection
Source: Insects. 2024 May 1;15(5):326. doi: 10.3390/insects15050326 (PMC11121787; doi:10.3390/insects15050326)

Table S1. The RNA-seq summary results of raw reads, clean reads, and values of Q20 and Q30 (*n* = 4)

Sample Name	Raw Reads	Clean Reads	Clean Reads (%)	Q20 (%)	Q30 (%)
C-ZF1-Liver	17,002,570	16,286,344	95.79	95.3	85.4
C-ZF2-Liver	11,245,170	10,786,796	95.92	95.3	85.6
C-ZF3-Liver	17,766,634	17,712,137	99.69	98.9	96.2
C-ZF4-Liver	18,964,911	18,903,974	99.68	98.7	95.6
**Average**	**16,244,821**	**15,922,313**	**97.77**	**97.05**	**90.69**
BSF-ZF1-Liver	13,048,085	12,572,707	96.36	95.6	86.3
BSF-ZF2-Liver	11,471,571	11,063,075	96.44	95.7	86.4
BSF-ZF3-Liver	17,780,487	17,722,270	99.67	98.8	95.8
BSF-ZF4-Liver	16,945,367	16,889,629	99.67	98.8	96.0
**Average**	**14,811,378**	**14,561,920**	**98.03**	**97.22**	**91.12**
C-ZF1-Intestine	13,794,645	13,225,119	95.87	95.4	85.8
C-ZF2-Intestine	13,037,556	12,488,743	95.79	95.3	85.6
C-ZF3-Intestine	17,407,256	17,349,635	99.67	98.8	96.1
C-ZF4-Intestine	17,559,334	17,508,898	99.71	98.9	96.5
**Average**	**15,449,698**	**15,143,099**	**97.76**	**97.13**	**90.98**
BSF-ZF1-Intestine	16,424,021	15,746,737	95.88	95.3	85.4
BSF-ZF2-Intestine	13,627,128	13,163,224	96.60	95.9	86.8
BSF-ZF3-Intestine	18,291,754	18,233,975	99.68	98.8	96.0
BSF-ZF4-Intestine	17,179,385	17,126,727	99.69	98.9	96.2
**Average**	**16,380,572**	**16,067,666**	**97.96**	**97.20**	**91.11**

C-ZF is zebrafish control group (fed by basal diet); BSF-ZF is the name for treatment group fed by dipterose-BSF contained diet (0.1 μg/g)

Table S2. The RNA-seq summary table of clean reads and mapping results ($n = 4$)

Sample Name	Clean Reads	Number of unmapped reads to the reference	Number of reads mapped to the reference*	Number of reads mapped to reference (multiple sites)
C-ZF1-Liver	16,286,344	631,910.15 (3.88%)	15,173,986.70 (93.17%)	480,447.15 (2.95%)
C-ZF2-Liver	10,786,796	365,672.38 (3.39%)	9,873,154.38 (91.53%)	547,969.24 (5.08%)
C-ZF3-Liver	17,712,137	425,091.29 (2.4%)	16,732,655.82 (94.47%)	554,389.89 (3.13%)
C-ZF4-Liver	18,903,974	368,627.49 (1.95%)	17,966,336.89 (95.04%)	567,119.22 (3%)
**Average**	**15,922,313**	**447,825.33 (2.91%)**	**14,936,533.45 (93.55%)**	**537,481.37 (3.54%)**
BSF-ZF1-Liver	12,572,707	442,559.29 (3.52%)	10,992,317.73 (87.43%)	1,137,829.98 (9.05%)
BSF-ZF2-Liver	11,063,075	417,077.93 (3.77%)	10,293,084.98 (93.04%)	351,805.79 (3.18%)
BSF-ZF3-Liver	17,722,270	395,206.62 (2.23%)	16,882,234.40 (95.26%)	444,828.98 (2.51%)
BSF-ZF4-Liver	16,889,629	454,331.02 (2.69%)	15,996,167.63 (94.71%)	439,130.35 (2.60%)
**Average**	**14,561,920**	**427,293.71 (3.05%)**	**13,540,951.18 (92.61%)**	**593,398.77 (4.34%)**
C-ZF1-Intestine	13,225,119	562,067.56 (4.25%)	12,218,687.44 (92.39%)	444,364.00 (3.36%)
C-ZF2-Intestine	12,488,743	494,554.22 (3.96%)	11,668,232.58 (93.43%)	325,956.19 (2.61%)
C-ZF3-Intestine	17,349,635	421,596.13 (2.43%)	16,513,382.59 (95.18%)	416,391.24 (2.40%)
C-ZF4-Intestine	17,508,898	418,462.66 (2.39%)	16,682,478.01 (95.28%)	409,708.21 (2.34%)
**Average**	**15,143,099**	**474,170.14 (3.26%)**	**14,270,695.16 (94.07%)**	**399,104.91 (2.68%)**
BSF-ZF1-Intestine	15,746,737	672,385.67 (4.27%)	14,428,735.11 (91.63%)	644,041.54 (4.09%)
BSF-ZF2-Intestine	13,163,224	541,008.51 (4.11%)	12,085,155.95 (91.81%)	535,743.22 (4.07%)
BSF-ZF3-Intestine	18,233,975	475,906.75 (2.61%)	17,243,870.16 (94.57%)	514,198.10 (2.82%)
BSF-ZF4-Intestine	17,126,727	385,351.36 (2.25%)	16,371,438.34 (95.59%)	369,937.30 (2.16%)
**Average**	**16,067,666**	**518,663.07 (3.31%)**	**15,032,299.89 (93.40%)**	**515,980.04 (3.29%)**

C-ZF is zebrafish control group (fed by basal diet); BSF-ZF is the treatment group fed by dipterose-BSF contained diet (0.1 μg/g). The average percentage number of reads mapped to the reference is 93.41%.
* The filtered data reads were mapped using STAR (ver. 2.17.11b) to the reference sequence of *Danio rerio* (GRCz11 (GCA_000002035.4)

Figure S1. MDS plot verifying biases between C-ZF (control group) as blue circle and BSF-ZF (dipterose-BSF dietary inclusion 0.1 µg/g) as green circle

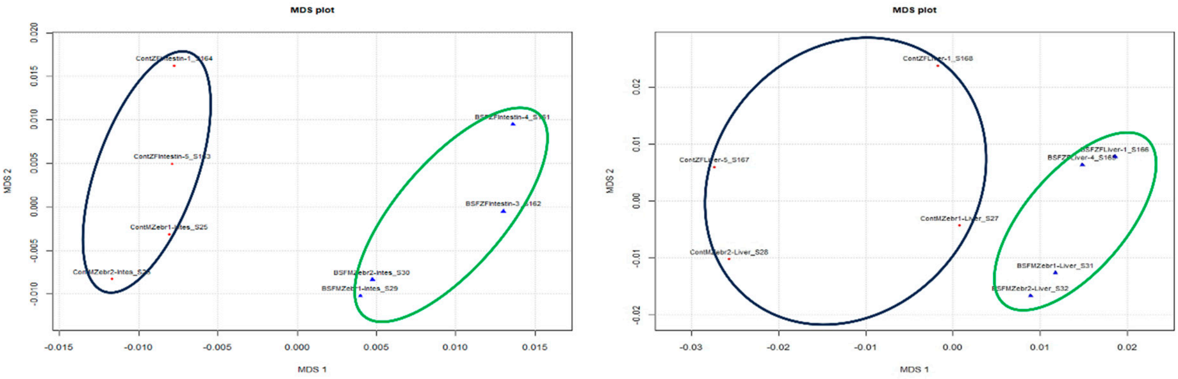

Figure S2. The heatmap display of DEGs obtained from zebrafish liver. Samples are shown in columns (n = 4), while DEGs are in rows.

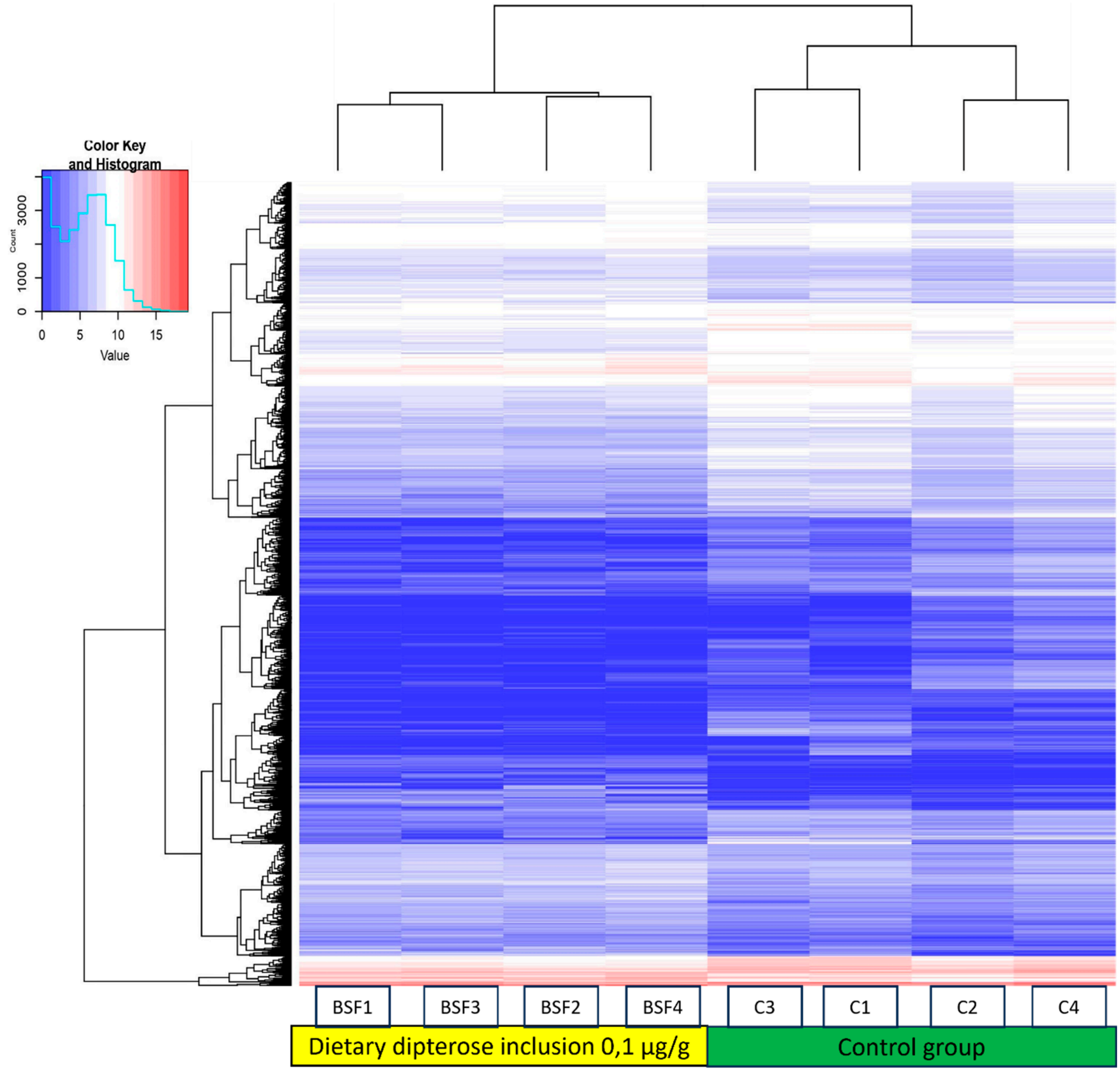

Figure S3. The heatmap display of DEGs was obtained from the zebrafish intestine. Samples are shown in columns (n = 4), while DEGs are in rows.

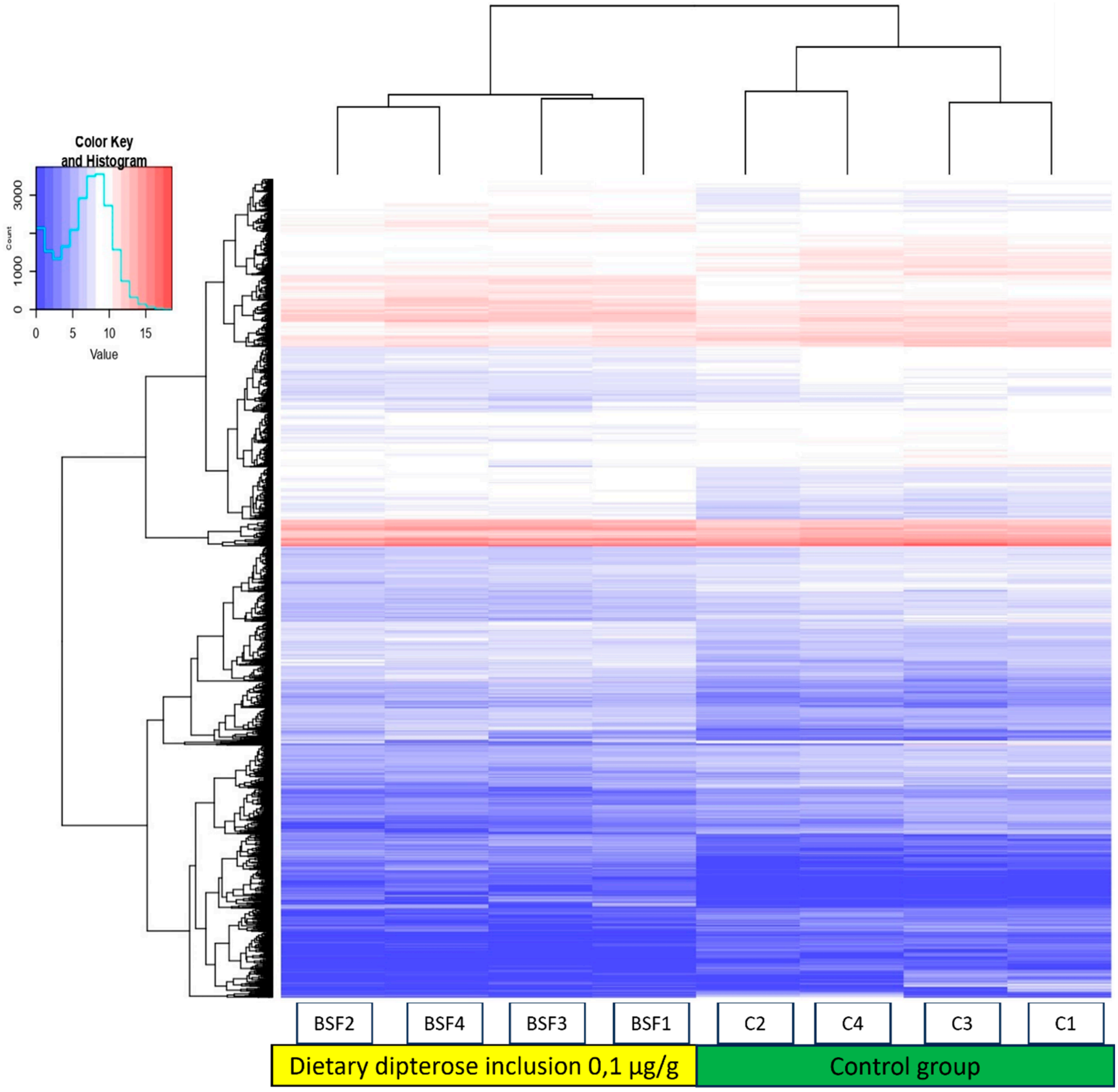

Supplement: Supplementary file 1 [file insects-15-00326-s001.zip › insects-2962467-supplementary.pdf]
